# Expression and localization of aquaporin 1b during oocyte development in the Japanese eel (*Anguilla japonica*)

**DOI:** 10.1186/1477-7827-9-71

**Published:** 2011-05-27

**Authors:** Hirohiko Kagawa, Takafumi Kishi, Koichiro Gen, Yukinori Kazeto, Ryota Tosaka, Hajime Matsubara, Takahiro Matsubara, Sayumi Sawaguchi

**Affiliations:** 1 Faculty of Agriculture, University of Miyazaki, Miyazaki 889-2192, Japan; 2 National Research Institute of Aquaculture, Fisheries Research Agency, Mie 519-0423, Japan; 3 Faculty of Bioindustry, Tokyo University of Agriculture, Hokkaido 099-2493, Japan; 4 South Ehime Fisheries Research Center, Ehime University, Ehime 798-4292, Japan; 5 Hokkaido National Fisheries Research Institute, Fisheries Research Agency, Hokkaido 085-0802, Japan

## Abstract

To elucidate the molecular mechanisms underling hydration during oocyte maturation, we characterized the structure of Japanese eel (*Anguilla japonica*) novel-water selective aquaporin 1 (AQP1b) that thought to be involved in oocyte hydration. The *aqp1b *cDNA encodes a 263 amino acid protein that includes the six potential transmembrane domains and two Asn-Pro-Ala motifs. Reverse transcription-polymerase chain reaction showed transcription of Japanese eel *aqp1b *in ovary and testis but not in the other tissues. *In situ *hybridization studies with the eel *aqp1b *cRNA probe revealed intense eel *aqp1b *signal in the oocytes at the perinucleolus stage and the signals became faint during the process of oocyte development. Light microscopic immunocytochemical analysis of ovary revealed that the Japanese eel AQP1b was expressed in the cytoplasm around the yolk globules which were located in the peripheral region of oocytes during the primary yolk globule stage; thereafter, the immunoreactivity was observed throughout the cytoplasm of oocyte as vitellogenesis progressed. The immunoreactivity became localized around the large membrane-limited yolk masses which were formed by the fusion of yolk globules during the oocyte maturation phase. These results together indicate that AQP1b, which is synthesized in the oocyte during the process of oocyte growth, is essential for mediating water uptake into eel oocytes.

## Background

Teleost oocytes are arrested at the prophase of the first meiotic division during their long period of growth (vitellogenic phase) [1]. After completion of vitellogenesis, oocytes undergo maturation (meiosis resumption) which is accompanied by several important processes, such as germinal vesicle breakdown (GVBD), hydration of oocytes, lipid coalescence and clearing of ooplasm [2]. In particular, in marine teleosts spawning buoyant eggs in seawater, oocytes undergo a significant increase in size because of rapid water uptake during meiosis resumption [3-5]. During these processes, the oocytes become buoyant, which is essential for their oceanic survival and dispersal as well as for the initiation of early embryogenesis [4,6,7].

Previous studies in marine teleosts producing buoyant (pelagic) eggs in seawater [8,9] and our recent study in the Japanese eel, *Anguilla japonica *[5] indicated that free amino acids and small peptides produced by yolk protein hydrolysis [6,7,10] and the accumulation of ions such as K^+ ^and Cl^- ^[10,11] during oocyte maturation provide an osmotic driving force for water influx into the oocyte. Moreover, aquaporin, an open molecular channel transporting water and other solutes along an osmotic gradient [12], was found to contribute to the rapid water influx into the oocyte during oocyte maturation in the gilthead seabream (*Sparus auratus*; [4,9]. In the Japanese eel [5], although possible contribution of aquaporin on oocyte hydration is suggested, no direct evidence have not been available until today.

Recent phylogenetic and genomic analyses showed that teleosts have two closely linked AQP1 paralogous genes, termed *aqp1a *and *aqp1b *(formally AQP1o). In the gilthead seabream, the *aqp1b *gene was highly expressed in the ovary containing previtellogenic and early vitellogenic oocytes [4]. Immunocytochemical analysis [4,7] revealed that AQP1b protein appeared to be located within a thin layer just below the oocyte plasma membrane. These observations therefore indicate that AQP1b is synthesized *de novo *by the oocyte at the initiation of vitellogenesis on an already existing mRNA pool. Closely linked to *aqp1b *paralogous gene, termed *aqp1a*, was also found from European eel kidney and was ubiquitously expressed [13]. Although there have been several other reports on the AQP contributing to osmoregulation in intestine [14], and gills [15], there is no detailed information concerning the AQP related mechanisms of oocyte hydration in eel.

Freshwater eels of the genus *Anguilla *are distributed worldwide and have unique characteristics such as a catadromous life history. The Japanese eel *A. japonica *is believed to migrate from the rivers into the ocean and spawn eggs in a particular area in the western North Pacific ocean (west of the Mariana Islands; [16]). Japanese eels found in rivers and the costal region of Japan are sexually immature and never mature under commercial rearing conditions [17,18]. Repeated injections of salmon pituitary extracts (SPE) induced vitellogenesis and subjective injection of 17, 20β-dihydroxy-4-pregnen-3-one (DHP, an eel maturation-inducing steroid) successfully induced maturation and ovulation of oocytes [18-21]. *In vitro *addition of DHP into the incubation medium also induced GVBD and ovulation of oocytes [5,22,23]. During i*n vivo *and *in vitro *maturation, Japanese eel oocytes undergo a significant increase in size because of rapid water uptake, and the eggs become buoyant [5]. Recent *in vitro *experiments from our laboratory showed that addition of HgCl_2_, an inhibitor of AQP water permeability, inhibited HCG- or DHP-induced water influx into oocytes and moreover, the inhibition was reversed by the addition of β-mercaptoethanol, suggesting that AQP facilitates water uptake by acting as a water channel in the oocyte of the Japanese eel [5]. However, there are no studies on AQP gene expression and its protein localization in the oocytes of Japanese eel or other primitive teleosts.

Therefore, in order to clarify AQP mediated mechanisms of oocyte hydration in the Japanese eel, the present paper reports the cloning, expression and sub-cellular localization of *aqp1b *using *in situ *hybridization and immunocytochemistry during oocyte growth and maturation.

## Methods

### Fish and ovarian samples

Cultured female Japanese eels weighing approximately 300 to 500 g were obtained from a fish farm and the Shibushi Station, National Center for Stock Enhancement, Fisheries Research Agency, Japan. After acclimation to seawater, they were kept without feeding in 400-L indoor circulating tanks under a natural photoperiod at a water temperature of 20°C. Cultured female eels are sexually immature and the oocytes never develop exceeding the early vitellogenic stage under the rearing condition [18,24]. To induce sexual maturation, they were intraperitoneally injected with SPE (20-30 mg/kg body weight) once a week. SPE was prepared by homogenizing salmon (*Oncorhynchus keta*) pituitary powder with a 0.9% NaCl solution, followed by centrifugation at 3000 rpm [19,24,25]. Oocytes at the previtellogenic and vitellogenic stages were taken from maturing female eels during the process of artificially induced sexual maturation. After 10-13 injections, full-grown oocytes and oocytes at the migratory nucleus stage were taken from the genital pore of fully matured female eels with a polyethylene cannula. Matured oocytes were obtained from female eels that were processed according to the method described previously [18,19,25]. Briefly, females that possessed oocytes over 750 μm in diameter at the migratory nucleus stage were injected with SPE (30 mg/kg body weight) as a priming dose, followed 24 h later by an intraperitoneal injection of DHP (2 μg/g body weight). All animal experiments were conducted in accordance with the University of Miyazaki guidelines and every effort was made to minimize the number of animals used and their suffering.

### Isolation of Japanese eel aqp1b

The ovary was collected from a maturing female Japanese eel, frozen in liquid nitrogen, and stored -80°C until used. Total RNA was extracted from ovary with TRIzol Reagent (Invitrogen, Carlsbad, CA, USA). The RNA was quantified on the basis of the absorbance at 260 nm and appeared to be non-degraded on a 1% (w/v) denaturing agarose gel containing formaldehyde and ethidium bromide. Complementary DNA was synthesized from 1 μg of total RNA using random hexamers and the Omniscript Reverese Transcriptase kit (Qiagen, Hilden Germany). After reverse transcription (RT), one-tenth of the RT reaction product was then mixed with 0.5 μM of each primer, 0.5 mM dNTPs and 1.25 U PrimeSTAR HS DNA polymerase (Takara Biomedicals, Tokyo, Japan) in a 50-μl reaction volume and amplified by a three-step PCR protocol as follows: 94°C for 2 min followed by 30 cycles of 94°C for 15 s, 52°C for 30 s, and 72°C for 1 min. A pair of degenerate primers based on the nucleotide sequence of other vertebrates were designed to clone the Japanese eel *aqp1b *cDNA (sense: 5'-TGGAGGRCNGTBCTDGCYGAGCT-3'; antisense: 5'-GCT GGDCCRAAWGWYCGAGCAGGGTT-3'; IUB group codes were used: R = A/G, Y = C/T, W = A/T, B = C/G/T, D = A/G/T, N = A/C/G/T). Sequence information from the partial cDNA clone was used to design gene-specific primers for 5'-RACE and 3'-RACE to clone the full-length eel *aqp1b*.

5 'RACE and 3 'RACE were carried out using the GeneRacer Kit (Invitrogen) according to the manufacturer's instructions. Briefly, 1 μg total RNA was dephosphorylated with calf intestinal alkaline phosphatase and decapped using tobacco acid pyrophosphatase (TAP). The GeneRacer RNA oligo was ligated to the TAP-treated mRNA with T4 RNA ligase and a cDNA template generated by reverse transcription using SuperScript II and the GeneRacer oligo dT primer. The 5'end of the eel *aqp1b *was amplified using the antisense primers 5'GSP-1 (5'-AAGCCTTGAGCCACGGCAACACC-3') and 5'GSP-2 (5'-AGCTCGGAACACGCTTATCTGACAGC-3') in combination with the GeneRacer 5' Primer and GeneRacer 5' Nested Primer from the 5'RACE. For 3'RACE, sense primers 3'GSP-1 (5'-AGCTGTCAGATAAGCGTGTTCCGAGC-3') and 3'GSP-2 (5'-AAGCTAAATGGTGTTGCCGTGGCTC-3') were used in combination with the GeneRacer 3' Primer and GeneRacer 3' Nested Primer to amplify the 3' end of eel AQP1b cDNA. The resultant DNA fragment was ligated and subcloned using TOPO-XL (Invitrogen), and sequenced. The cDNA clones were sequenced using an ABI PRISM 3130 × l DNA sequencer (Applied Biosystems, Foster City, CA, USA). The nucleotide sequence was determined by analyzing more than four clones from distinct amplification to avoid PCR errors. The nucleotide and amino acid sequences were analyzed using the SeqMan software (DNAStar, Madison WI, USA) and the BLAST network service of the National Center for Biotechnology Information. The accession number of the sequence reported in this paper has been deposited in the DDBJ/EMBL/GenBank as **AB586029**.

### Phylogenetic analysis

Multiple alignments of deduced amino acid sequences were performed with CLUSTAL W [26]. A phylogenetic tree was constructed based on amino acid sequence alignments by the Neighbor-Joining method [27]. TreeView was used to display the phylogenetic tree. The degree of support for internal branches was further assessed by bootstrapping analysis consisting of 1000 replicates. All of the above analyses were performed with the Internet server of the DNA Data Bank of Japan [28]. Full species names and GenBank accession numbers of the species used to generate the tree are as follows: Japanese eel *Anguilla japonica *AQP1a, AB094502; European eel *Anguilla anguilla *AQP1a, AJ564420; AQP1b, EF011738; gilthead seabream *Sparus aurata *AQP1a, AY626939; AQP1b, AY626938; European seabass *Dicentrarchus labrax *AQP1a, DQ924529; Atlantic salmon *Salmo salar *AQP1a, ACI33306; zebrafish *Danio rerio *AQP1a, DQ887675; AQP1b, EU327345; mummichog *Fundulus heteroclitu*s AQP1a, EU780153; Senegalese sole *Solea senegalensis *AQP1b, AY626941; African clawed frog *Xenopus laevis *AQP1 BC072092; marine toad *Bufo marinus *AQP1a, U56603; AQP1b, 2206276A; chicken *Gallus gallus *AQP1, AM183252; rat *Rattus norvegicus *AQP1, BC090068; human *Homo sapiens *AQP1, NP_932766.

### Tissue distribution of messenger RNA

One microgram of total RNA was reverse-transcribed to first-strand cDNA using a random hexamer primer and Omniscript RT kit (Qiagen, Chatsworth, CA, USA). After reverse transcription (RT), one-tenth of the RT reaction product was then mixed with 0.5 μM of each primer, 0.5 mM dNTPs and 0.2 U PrimeSTAR HS DNA polymerase (Takara Biomedicals, Tokyo, Japan) in a 20-μl reaction volume and amplified by a three-step PCR protocol as follows: 94°C for 2 min followed by 30 cycles of 94°C for 30 s, 60°C for 30 s, and 72°C for 1 min. Primers specific to the Japanese eel *aqp1b *were designed, *aqp1b *sense (5'-GATTACCCTGGCTACGCTCATT-3') and *aqp1b *antisense (5'-CTTGAGCCACAGCAACACCA-3'). These primers flank an intron in the *aqp1b *sequence, so any amplicons from possible genomic contamination can be eliminated. A DNA positive control reaction was performed using primers for *β-actin *(GenBank accession number AB074846). Additionally, negative control reactions were performed using RNA (without RT) as a template in our amplification protocol. Ten microlitres of product from each reaction was analyzed using 2% agaraose gel in 1 × TAB stained with 0.5 μg/ml ethidium bromide. The amplified fragments of *aqp1b *and *β-actin*, 173 and 226 bp, respectively, were sequenced to verify their specification.

### In situ hybridization

To generate digoxigenin (DIG)-labeled antisense and sense RNA probes, cDNA was amplified by PCR using primers for eel *aqp1b*. The gene-specific downstream-primer contained an artificially introduced T7 RNA polymerase recognition sequence (5'-TAATACGACTCACTATA-3'), and a 6 bp transcription initiation sequence at its 5'-end to enable synthesis of transcripts. Primer sequences are shown below, whereby the underline indicates the promoter sequence: for generation of antisense probe: *aqp1b *forward: 5'-CATATTCGTTGGTATTTCAGCTGCAGTCGG-3'; *aqp1b *T7- reverse: 5'-TAATACGACTCACTATAGGGAGGGGAGGTAGTCATAGACAAGGGCAGCTACCA G-3'. For production of sense probe: *aqp1b *T7-forward: 5'-TAATACGACTCACTATAGGGAGG CATATTCGTTGGTATTTCAGCTGCAGTCGG-3'; *aqp1b *reverse: 5'-GGAGGTAGTCATAGACAA GGGCAGCTACCAG-3'. DIG-labelled RNA probes were generated from RT-PCR-derived templates by *in vitro *transcription performed using DIG RNA Labeling kit (Roche Diagnostics, Mannheim, Germany). The RNA probes synthesized were quantified and an equal amount of each probe was used for hybridization. *In situ *hybridizations were performed using DIG-labeled antisense and sense cRNA probes according to a slightly modified method of [29]. Briefly, freshly dissected ovarian fragments were fixed in Bouin's solution at 4°C overnight. Fixed ovarian fragments were embedded in paraffin, sectioned (5 μm) mounted onto silane-coated slides (Matsunami Glass, Osaka, Japan). The sections were deparaffinized, hydrated, and then hybridized with cRNA probes. Coverslips were placed over the sections, and the slides were incubated in humidified chambers at 58°C for 18 h. Hybridized probes were detected using the DIG Nucleic Acid Detection Kit (Roche Diagnostics).

### Antibody

**Figure 1:**
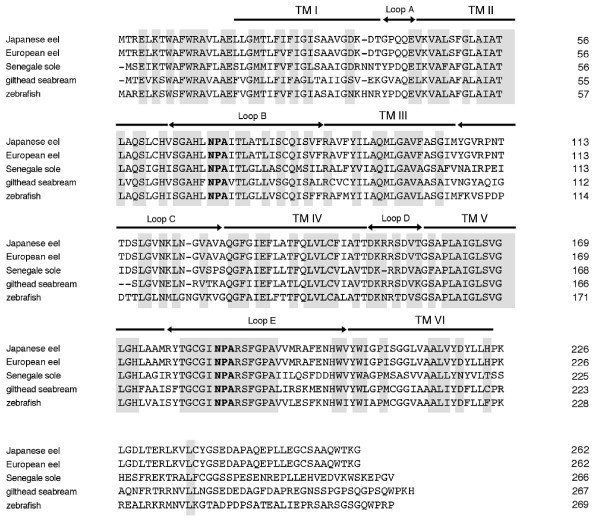
**Comparison of the deduced amino acid sequence of Japanese eel AQP1b with those of European eel, Senegal sole, gilthead seabream and zebrafish**. The six transmembrane (TM) domains and connecting loops (A-E) are indicated by brackets and horizontal arrows, respectively. Two Asn-Pro-Ala (NPA) motifs are emboldened. The antibody was raised against a synthetic peptide corresponding to the underlined sequence. The numbers on the right correspond to amino acid positions in the protein sequences.

A polyclonal antibody was raised in a rabbit against a synthetic peptide corresponding to part of the C-terminal region of eel AQP1b molecules (APAQEPLLEGCSAAQWTKG) (Figure 1). The antigen conjugated with keyhole limpet hemocyanin (KLM) was emulsified with complete Freund's adjuvant, and immunization was performed in a New Zealand white rabbit (Uniqtech Co. Ltd., Chiba, Japan). The antisera were affinity-purified on thiopropyl sepharose 6B coupled to the synthetic peptide. The anti-eel AQP1b antibody was used at 1:1000-2000 for immunocytochemistry and Western blot analysis.

### Western blot analysis

Ovarian fragments containing vitellogeinc oocytes at the various developmental stages were used in the experiments. Western blot analysis was performed according to the method of [30]. Prior to Western blotting, the ovarian fragments and oocytes were homogenized in SDS-PAGE sample buffer (10% SDS 4.5 ml: glycerin 3 ml: 2-mercaptoethanol 1.5 ml: bromophenol blue 0.75 ml: distilled water 0.25 ml) and the homogenates were centrifuged at 10,000 g for 40 min at 4°C. The supernatant was used for the following sodium-dodecyl-sulphate-polyacrylamide gel electrophoresis (SDS-PAGE). SDS-PAGE was performed on precast polyacryllaide gel with a gradient of total acrylamide concentrations from 5% to 20% (ATTO, Tokyo, Japan). Protein bands on SDS-PAGE gels were transblotted onto a Polyvinylidene difluoride (PVDF) membranes (Immobilon-P, Millipore, Bedford, MA) using a semidry transfer apparatus (Trans-Blot SD; Bio-Rad, Hercules, CA). The transferred proteins were stained with Coomassie brilliant blue (CBB), destained in 50% methanol and 5% acetic acid, washed with Milli-Q water (Millipore Corp.) and dried. Immunological detection of eel AQP1b was carried out using an antibody to the Japanese eel AQP1b described above. Immunoreactive proteins were visualized by using a Histofine streptoavidin-biotin peroxidase complex kit (Nichirei Co. Ltd., Japan).

### Immunocytochemistry

Ovarian fragments obtained from female eels were fixed in Bouin's solution at 4°C overnight. The immuocytochemical staining was performed as per the method described previously [31]. Briefly, deparaffinized sections were immersed in 3% hydrogen peroxide for 10 min to remove endogenous perxoidase staining. Immunoreactive proteins were visualized by using a Histofine streptoavidin-biotin peroxidase complex kit (Nichirei Co. Ltd., Japan). After incubation with 10% normal goat serum for 10 min, the sections were incubated with the primary antisera for 30 min. The sections were then rinsed in PBS and incubated with biotinylated anti-rabbit IgG for 10 min. After rinsing with PBS, the sections were incubated with streptavidin-linked horseradish-peroxidase (HRP) for 5 min. After a final wash with PBS, the HRP complex was developed with a solution of 0.001% hydrogen peroxidase-0.05% 3,3-diaminobezidine tetrahydrocholoride (Sigma, CA) in 0.05 M Tris-HCl buffer (pH 7.6). After immunostaining, the sections were counterstained with Mayer's hematoxylin. All procedures were conducted at room temperature. The specificity of the immunostaining was confirmed by the following controls: (1) primary antisera were substituted with normal rabbit serum (NRS) or PBS, (2) primary antisera were absorbed with purified synthetic peptide corresponding to part of the C-terminal region of eel AQP1b molecules. Replacement of primary antiserum with PBS or NRS abolished the immunostaining of the antiserum. The preabsorption of antiserum with an excess amount of the synthetic peptide also greatly reduced the immunostaining.

## Results

### Cloning of aquaporin1b cDNA and deduced amino acid sequence (Figure 1)

**Figure 2:**
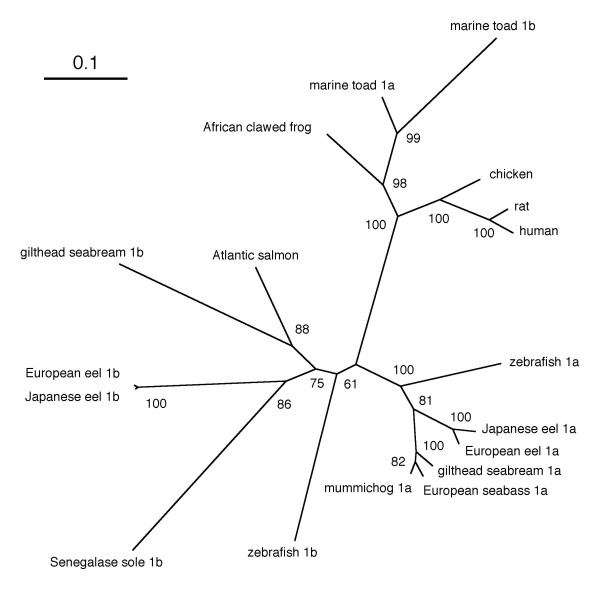
**Phylogenetic tree of vertebrate AQP1 proteins**. The unrooted phylogenetic tree was constructed by neighbor-joining method after alignment of deduced amino acid sequences of AQP1 protein. Values at interior nodes are bootstrap percentages derived from 1000 replications. The scale bar indicates an evolutionary distance of 0.1 amino acid substitution per position in the sequence.

The deduced protein sequence has 263 amino acids with a calculated molecular mass of 28.0 kDa. According to Kyte-Doolittle hydropathic analysis, AQP1b contained six hydrophobic putative transmembrane domains connected by five loops (A-E) and extracellular N-terminal and cytoplasmic C-terminal domains. Further analysis indicated that Japanese eel AQP1b had a Asn-Pro-Ala (NPA) motif in loops B and E, which is the hallmark of the membrane intrinsic protein family. Figure 1 shows the deduced amino acid sequence of the Japanese eel *aqp1b *cDNA aligned with counterparts from other species. Japanese eel AQP1b shared high homology with European eel (99%), zebrafish (65%), Senegale sole (62%), gilthead seabream (61%) AQP1b, with lower identity to AQP1 from human (54%). The deduced amino acid sequence homology of AQP1b with teleost AQP1s was as follows: Japanese eel AQP1a 64%; European eel AQP1a 64%; gilthead seabream AQP1a 66%; zebrafish AQP1a 61%; Xenopus AQP1 55%; chicken AQP1 55%; rat AQP1 53%; human AQP1 54%. To illustrate the phylogenetic relatedness of the Japanese eel AQP1b with other reported AQP proteins, an evolutionary tree was constructed using the neighbor-joining method of CLUSTAL W. As expected from the sequence alignment, Japanese eel AQP1b fell into the cluster with the AQP1b subfamily from other species (Figure 2).

### Eel AQP1b expression and tissue distribution (Figure 3)

**Figure 3:**
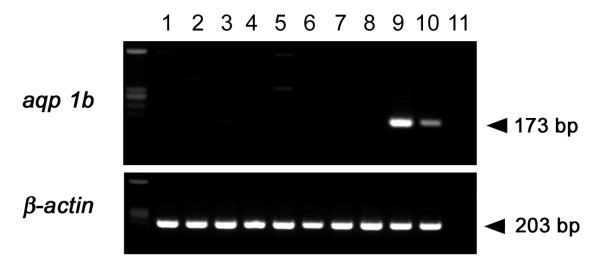
**Tissue distribution of *aqp1b *in Japanese eel**. RT-PCR was performed using 1 μg of total RNA prepared from Japanese eel tissues using the Omniscript kit (Qiagen). Amplification products were analyzed on a 2.0% agarose gel and stained by ethidium bromide. Control RT-PCR of β-actin using the same amount of cDNA is also indicated in the lower panel. Lane from the left is brain(1), eye(2), gill(3), esophagus(4), heart(5), liver(6), kidney(7), intestine(8), ovary(9), testis(10), and no template (11), respectively.

The expression of ovary-derived *aqp1b *in different Japanese eel tissues was assessed by RT-PCR analysis. For RT-PCR analysis, selected regions of AQP1b cDNA were amplified from the RT mixture with sequence-specific primers. As shown in Figure 3, an abundant amplification product of the *aqp1b *transcript was detected in Japanese eel ovary and a relatively lower amount in the testis. In contrast, the expression was barely detectable in the other tissues tested (brain, eye, gill, oesophagus, heart, liver, kidney, small intestine, and muscle). We repeated the analysis using different primer sets and found similar results (data not shown).

### In situ hybridization

**Figure 4:**
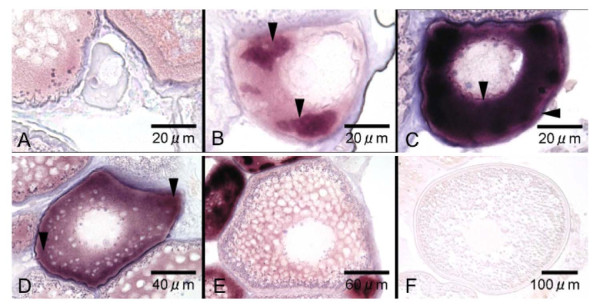
**Localization of *aqpb1 *gene transcripts in Japanese eel oocytes by *in situ hybridization***. Oocyte at the perinucleolus stage (A, B, C), the oil droplet stage (D), the primary yolk globule stage (E), and the secondary yolk globule stage (F). Arrows indicate intense AQP1b signals observed in oocytes.

*In situ *hybridization was carried out to characterize the cellular expression of eel *aqp1b *in the oocytes at various developmental stages. Although an *aqp1b *signals could not be observed at oocytes at the perinucleolus stage (Figure 4A), the intense signal was first found in larger oocytes at the same stage (Figure 4B). Relatively intense signals were localized to regions within the cytoplasm (Figure 4B) and later they became distributed throughout the cytoplasm (Figure 4C). Similar intense signals were found in the oocyte at the oil droplet stages (Figure 4D). Thereafter, the expression of eel *aqp1b *signals became weak in association with oocyte growth from the primary yolk (Figure 4E) to the secondary yolk globule stage (Figure 4F) and sometimes became absent.

### Immunocytochemistry

**Figure 5:**
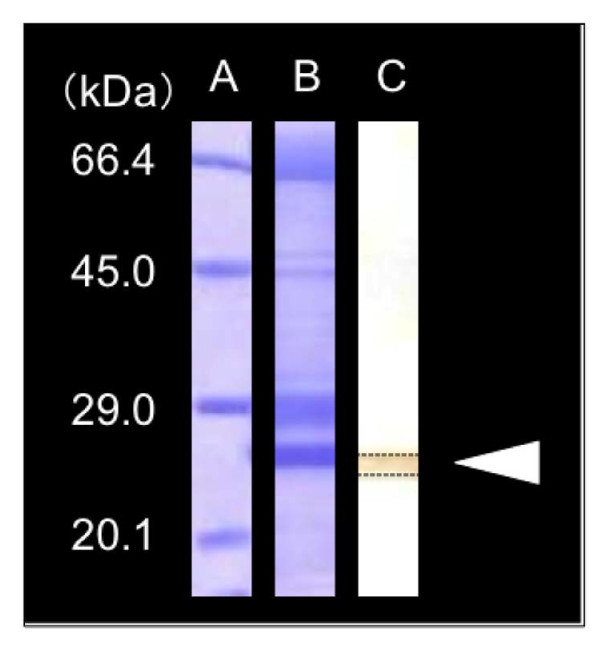
**Electrophoretic and immunological analysis of AQP1b in the Japanese eel ovarian fragments**. SDS-PAGE stained with Coomassie blue (lane B) and corresponding Western blot of protein extracts from ovarian fragments using anti-eel AQP1b antiserum (lane C). Molecular mass values are provided on the left (lane A) in kDa. Arrow head indicates the immunoreaction of AQP-1b.

**Figure 6:**
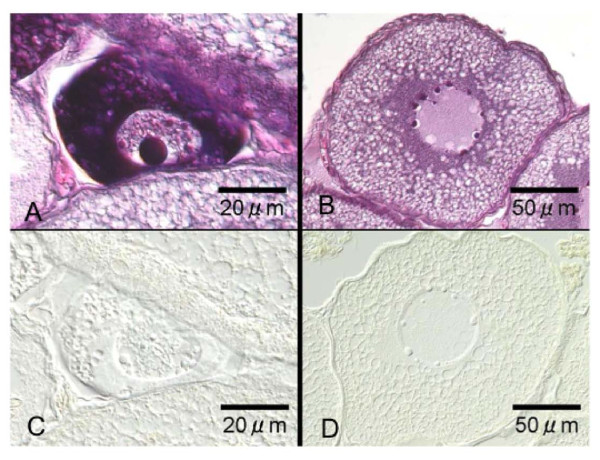
**Immunohistochemical localization of AQP1b in Japanese eel perinucleolus and oil droplet stage oocytes**. Two successive sections of the Japanese eel oocytes at the perinucleolus stage (A, C) and at the oil droplet stage (B, D) stained with hematoxylin and eosin (A, B) and stained with immunocytochemically with anti-eel AQP1b (C, D). AQP1b immunoreaction was not detected in the oocytes at these stages.

**Figure 7:**
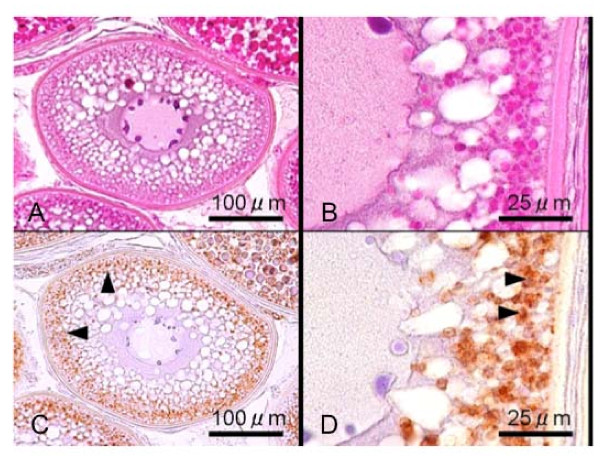
**Immunohistochemical localization of AQP1b in Japanese eel primary yolk globule oocytes**. Two successive sections of the Japanese eel oocytes at the primary yolk globule stage stained with hematoxylin and eosin (A, B) and immunocytochemically stained with anti-eel AQP1b (C, D). Intense AQP1b immunoreaction was detected around yolk granules located in the peripheral region of oocyte (B, D).

**Figure 8:**
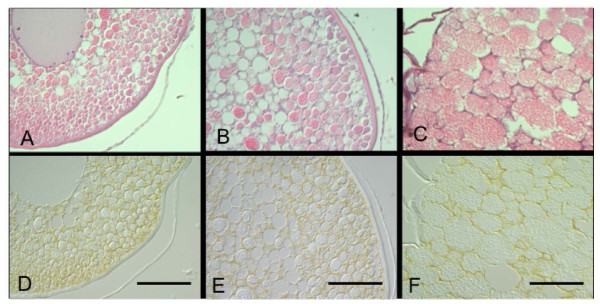
**Immunohistochemical localization of AQP1b in Japanese eel tertiary yolk globule, migratory nucleus, and mature stage**. Two successive sections of the Japanese eel oocytes at the tertiary yolk globule stage (A, D), the migratory nucleus stage (B, E), and the mature stage (C, F) stained with hematoxylin and eosin (A, B, C) and immunocytochemically stained with anti-eel AQP1b (D, E, F). Faint but clear immunoreaction was detected around the yolk globules (D) and the large yolk mass (E, F). Bars indicate 50 μm.

Immunoblotting analysis using the eel AQP1b antisera on the extracts from ovarian fragments showed a single protein band with a molecular mass of approximately 28 kDa, thus corresponding to the calculated molecular mass of Japanese eel AQP1b (Figure 5). Immunocytochemical observation for eel AQP1b was performed in ovarian fragments containing oocytes at various developmental stages. Previtellogenic ovarian follicles at the perinucleolus and oil droplet stages were devoid of eel AQP1b positive reaction (Figure 6). Intense immunoreaction was first observed in oocytes at the primary yolk globule stage, exclusively in the vesicles (which are surmise yolk globules) located at the peripheral ooplasm (Figure 7). Full-grown oocytes at the tertiary yolk globule stage showed the germinal vesicle in a central position and small yolk globules distributed in the cytoplasm. Intense immunoreactions were located around yolk globules occupied in oocyte cytoplasm (Figure 8A, D). During oocyte maturation phase, yolk globules fused together and increased in size in oocytes at the migratory nucleus stage (Figure 8B) and yolk globules became larger yolk masses but they did not fuse into a single yolk mass in oocytes at the mature stage (Figure 8C). Immunoreactions were observed around the membrane bound yolk masses in oocytes at the migratory nucleus stage (Figure 8E) and at the mature stage (Figure 8F).

## Discussion

We have isolated and characterized a Japanese eel *aqp1b *cDNA derived from ovary. The predicted amino acid sequences of the cloned Japanese eel ovary-derived *aqp1b *shared 99% overall sequence identity with that of the AQP1 previously reported in the European eel, *Anguilla anguilla *[13] termed AQP1dup. Tingaud-Sequeira et al. [32] indicated, from results of phylogenetic and genomic analyses, that teleosts, unlike tetrapods, have two closely linked *aqp1b *paralogous genes, termed *aqp1a *and *aqp1b*. European *AQP1dup *is identical to that recently isolated from the same species and named *aqp1b*. Therefore, it is certain that the Japanese eel AQP that we cloned from the ovary is a homologue of *aqp1b*. The Japanease eel APQ1b contains three functional domains; an N-terminal extracellular domain, a large transmembrane domain, and a C-terminal cytoplasmic domain. In particular, six potential transmembrane domains and two NPA motifs are conserved. Moreover, amino acids known to be essential for the pore-forming region in human AQP1 (i.e., Phe^56^, His^180^, and Arg^195 ^[33]) were present in an analogous position in Japanese eel AQP1b. Therefore, these amino acids in Japanese eel AQP1b may be involved in water-selective pore formation. Also, a Cys residue located N-terminal to the second NPA motif, which may be involved with inhibition of water permeability by mercurial compounds [34], was identical in Japanese eel AQP1b. In silico analysis of AQP1b C-terminal amino acid sequence revealed that a consensus residue (Ser), had high phosphorylation score and fulfilled the criteria for a Pro-directed kinase phosphorylation site, also found in the C-terminus of fugu, sole, and sea bream AQP1b, but not European eel [32]. These consensus sites may be involved in the control of AQP1b intracellular trafficking through phosphorylation-independent and -dependent mechanisms [32].

Present RT-PCR analysis revealed that Japanese eel *aqp1b *transcripts were highly abundant in the ovary. The present result observed in the ovary is in accordance with the previous studies in teleosts spawning pelagic eggs into seawater, such as the European eel, gilthead seabream and Senegalese sole [32]. Our data also showed faint, but significant, *aqp1b *transcript expression in the testis of Japanese eel, however expression was not detected in other tissues. Although relatively lower levels of *aqp1b *transcripts were found in gut, kidney and gills of gilthead seabream and the European eel [32], specific *aqp1b *transcripts were not observed in testis. These differences in the AQP1b distribution, especially that of testis, may reflect differences between species and/or sexual maturation stages, although the exact reasons are unclear, as detailed physiological information about the stage of fish was not provided in the previous report [32]. In mammals, AQPs are involved in the regulation of fluid resorption in the efferent duct [35,36] and also in the volume reduction of spermatids [37]. Therefore, in teleosts, further investigation of the physiological significance of AQP in testis is warranted.

*In situ hybridization *analysis showed that eel *aqp1b *mRNA was expressed in oocytes at the perinucleolus stage. This is the first information showing sub-cellular localization of *aqp1b *gene transcripts in any teleost oocytes and the result confirms the RT-PCR data indicating that seabream *aqp1b *is expressed in ovaries containing oocytes at the previtellogenic and early vitellogenic stages. Moreover, our data showed the dispersion of intense hybridization signals found in oocytes at the perinucleolus stage become faint in association with oocyte growth and maturation. The previous data obtained by RT-PCR showed that the levels of seabream *aqp1b *mRNA did not change significantly during oocyte growth and maturation [4]. Therefore, in Japanese eel, as indicated in seabream [4], mRNA which is synthesized in oocytes during the early growth phase might be dispersed in oocyte cytoplasm but total amounts of *aqp1b *mRNA might maintain constant levels during subsequent oocyte growth and maturational stages. RT-PCR analysis is needed to obtain direct evidence on changes of aqp1b mRNA levels during oocyte maturation of Japanese eel in future.

Immunocytochemical analysis showed that eel AQP1b protein is synthesized in early vitellogenic oocytes. These results are identical to the data obtained in oocytes of gilthead seabream [4]. During oocytes growth, gonadotropin, follicle-stimulating hormone (FSH) [38], directly acts on ovarian follicles to produce estradiol-17β (E_2 _) through the collaborative actions of theca and granulosa cell layers [39]. Vitellogenin is synthesized by hepatocytes following induction by E_2 _[40] and is then incorporated into growing oocytes by receptor-mediated endocytosis [41,42] by stimulation of FSH [43,44]. Therefore, FSH, E_2 _or other factors, such as IGF-I [31] found in the follicles, may regulate eel AQP1b synthesis at post-translational levels, as suggested in the previous studies of mammals [45,46].

During oocyte maturation of Japanese eel (meiosis resumption), the yolk granules fused and increased in size to become large yolk masses but did not form a single yolk mass [5]. These morphological changes observed during oocyte maturation is different from those observed in the gilthead seabream [4] in which the yolk granules fuse into a single yolk mass. In the gilthead seabream, during oocyte maturation, AQP1b translocated towards oocyte periphery and become concentrated within a thin layer just below the oocyte plasma membrane, suggesting that AQP1b located at the plasma membrane is essential for water influx into oocytes. However, in the Japanese eel, immunocytochemical analysis showed that immunoreactions of eel AQP1b were mainly observed around the fused yolk masses in oocytes at the migratory nucleus and mature stages. Localization of AQP1b just below the oocyte plasma membrane found in the gilthead seabream is not apparent in the present study. These morphological differences of yolk masses and different localization of AQP1b during oocyte maturation observed between gilthead seabream and the Japanese eel may be reflected by the varied mechanism of hydration of oocytes. In the Japanese eel, oocyte hydration may be regulated by a two-step mechanism. At the start of hydration, water may pass into the oocyte through the plasma membrane and then into the yolk mass through AQP1b localized in the yolk membrane, resulting in swelling of the yolk mass. As suggested in the gilthead seabream [4], it may also be possible that water influx from blood and ovarian fluid into oocyte can occur by simple diffusion (a comparatively slow influx) through the follicular (somatic cells) and oocyte membranes, since a longer period of time (5 days *in vivo *[5]) is required to accomplish hydration during oocyte maturation of the Japanese eel. Further studies are necessary to obtain conclusive evidence of AQP1b localization on plasma membrane of Japanese eel oocytes.

## Conclusions

Based on the present study and the previous *in vitro *data [5], mechanisms of oocyte hydration during meiotic maturation can be explained in the Japanese eel. During the previtellogenic stage (at the perinucleolus stage), mRNA of eel *aqp1b *are synthesized in the oocytes, perhaps by maternal gene expression and/or synthesized from ovarian follicle, since the darkly staining aqp1 transcript masses are found around oocytes. Synthesis of AQP1b protein is stimulated when oocytes begin vitellogenesis. The gonadotropin, FSH and/or E_2 _may be involved in the initiation of AQP1b synthesis in oocytes. During oocyte maturation, accumulated AQP1b in ooplasm is translocated around the yolk mass, which forms by the fusion of yolk globules, and by the proteolytic cleavage of vitellogenin-derived yolk proteins. Water transport is mediated by these AQP1bs which are located at different sites on the oocytes along with the osmotic driving force created by the accumulated yolk protein-derived free amino acids and inorganic ions [5]. Further studies on other evolutionally primitive species, such as conger eel *Conger myriaster *and Pike eel *Muraenesox cinereus*, may provide more insight to confirm this contention.

## Competing interests

The authors declare that they have no competing interests.

## Authors' contributions

HK conceived and designed the study. HK and KG wrote MS and discussed. TF, KG, HM, RT, and YK performed molecular experiments. TM and SS participated in the protein analysis. All authors read and approved the final manuscript.
